# Colostrum Replacer and Bovine Leukemia Virus Seropositivity in Calves

**DOI:** 10.3201/eid1906.121523

**Published:** 2013-06

**Authors:** Bhudipa Choudhury, Christopher Finnegan, Jean-Pierre Frossard, Chris Venables, Falko Steinbach

**Affiliations:** Animal Health and Veterinary Laboratories Agency, New Haw, Surrey, UK

**Keywords:** bovine leukemia virus, enzootic bovine leukosis, serology, colostrum, viruses

**To the Editor:** Bovine leukemia virus (BLV), a deltaretrovirus in the family *Retroviridae*, is the causative agent of enzootic bovine leukosis (EBL). Although EBL is still endemic to the Americas and eastern Europe, most countries in western Europe are EBL free in accordance with European Union legislation; for example, Great Britain has held EBL-free status since 1999. EBL is notifiable to the World Organisation for Animal Health (*1*), with disease incursion affecting international trade. Infection with BLV is life-long and persistent; the presence of antibodies or integrated proviral DNA are indicators of virus exposure. Two tests prescribed for international trade are the agar gel immunodiffusion test (AGIDT) and ELISA (*1*); these tests are used widely for diagnosis (*2*). 

We report 5 cases that occurred in the United Kingdom during 2009, in which calves became seropositive for BLV after consuming colostrum replacer. In Dumfriesshire, Scotland, routine serologic screening for BLV detected seropositivity in 2 calves, which were artificial insemination bull candidates. In Newport, Wales, a BLV-seropositive calf was identified during pre-export testing. And in Yorkshire, England, 2 more BLV-seropositive calves, also artificial insemination bull candidates, were identified. All calves were home bred, and there was no evidence (as documented by serologic testing) or history of EBL within the herd. The farms were considered to have low risk for disease incursion because the introduction of new animals was limited. 

Further inquiry revealed that the calves had each been exclusively fed a colostrum replacer from North America, where BLV is endemic. Antibodies to BLV might have been present in the colostrum replacer and thus passively acquired by the calves, resulting in seropositivity. 

The hypothesis was tested by monthly blood sampling and ELISA analysis for antibodies against BLV (Institute Pourquier, Montpellier, France). Although the batch of colostrum replacer that had been fed to the calves from Dumfriesshire was not available for investigation, another colostrum sample was obtained from the same manufacturer for analysis. The reconstituted colostrum replacer was tested by AGIDT (IDEXX, Bern, Switzerland) at the following dilutions: neat (manufacturer’s guidelines), 1:2, 1:4, and 1:8. In addition, 2 commercial ELISA tests (Institute Pourquier and IDEXX) were used over a series of dilutions to 1:125. All serologic tests were conducted according to manufacturer’s recommendations. To examine the samples for proviral DNA, we conducted PCRs to amplify a 385-bp fragment of the envelope gene (*3*).

At the various dilutions of colostrum replacer, all serologic tests gave clearly positive reactions. Proviral PCR of the colostrum replacer also returned positive results, which were confirmed by sequencing. The resultant envelope sequence (GenBank accession no. HF545344) was aligned with 23 other sequences obtained from GenBank, which encompassed all known BLV genotypes. Phylogenetic analysis was conducted as described (*4*) and revealed clustering within genotype 1, which is consistent with BLV of North American origin (*4*). The hypothesis that colostrum intake had caused the seropositivity was supported by the declining antibody titers found in serial blood sampling of all 5 calves (Table).

**Table:** Bovine leukemia virus seropositivity of calves fed colostrum replacer, UK, 2009*

Location	Time from initial sampling, mo				
	0	1	2	3	4
Dumfriesshire, Scotland					
Calf 1	+++	++	+	IC	–
Calf 2	+++	+	+	–	–
Newport, Wales					
Calf 1	++	++	NS	NS	–
Yorkshire, England					
Calf 1	++	+	+	NS	–
Calf 2	++	+	+	NS	–

*Tested by ELISA (Institute Pourquier, Montpellier, France). +++, strong positive, sample to positive (S/P) ratio >200; ++, positive, S/P ratio >100; +: weak positive, S/P ratio 60–99; –, negative, S/P ratio <60; IC, inconclusive, S/P ratio 60–70; NS, not sampled.

The same brand of colostrum replacer was used on all 3 farms. For the farms in Wales and England, it was possible to sample the batch of colostrum powder being used; aliquots from each farm were BLV positive by AGIDT and ELISA.

Reactions to passively acquired antibodies would be expected to decrease and become undetectable. After exposure to virus and subsequent infection, antibody titers would not wane to undetectable levels. Our results (Table) provide evidence that the serologic reactions reported here resulted from ingestion of the colostrum replacer rather than BLV infection. The policy and international trade implications of such cases for Great Britain have been discussed (*5*). To maintain the disease-free status of the country, it was necessary to follow up with these cases, which inconvenienced farmers because of movement restrictions and, consequently, financial loss.

The cases described were all linked by the brand of colostrum used; however, our additional investigations found that other brands also tested BLV positive by AGIDT and thus could cause an effect similar to that described here. These data would therefore be useful to any organization involved in BLV serologic surveillance. As a result of this investigation, in March 2010 the European Union banned the import of calf colostrum from herds that are not EBL free.

Although PCR confirmed the presence of BLV proviral DNA in the colostrum, detection of such does not mean that the colostrum contained viable virus. Retroviruses, including BLV, are heat labile; thus, it is unlikely that viable BLV would survive the spray-drying production process. Whether other agents can survive warrants further investigation.
